# 4-Methyl-*N*-*p*-tolyl­piperidine-1-carbox­amide

**DOI:** 10.1107/S1600536812016248

**Published:** 2012-04-21

**Authors:** Yu-Feng Li

**Affiliations:** a Microscale Science Institute, Department of Chemistry and Chemical Engineering, Weifang University, Weifang 261061, People’s Republic of China

## Abstract

In the title mol­ecule, C_14_H_20_N_2_O, the piperidine ring has a chair conformation and its N atom is close to planar (bond-angle sum = 357.5°). The dihedral angle between the amide group and the aromatic ring is 47.43 (19)°. In the crystal, mol­ecules are linked into [100] *C*(4) chains by N—H⋯O hydrogen bonds.

## Related literature

For the medicinal properties of related compounds, see: Yang *et al.* (1997 ▶). For a related structure, see: Li (2011 ▶).
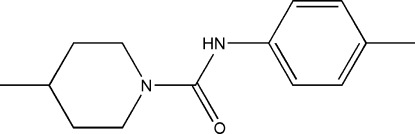


## Experimental

### 

#### Crystal data

C_14_H_20_N_2_O
*M* *_r_* = 232.32
Orthorhombic, 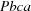

*a* = 9.6192 (19) Å
*b* = 11.127 (2) Å
*c* = 26.574 (5) Å
*V* = 2844.3 (9) Å^3^
*Z* = 8
Mo *K*α radiation
μ = 0.07 mm^−1^
*T* = 293 K
0.25 × 0.20 × 0.18 mm

#### Data collection

Bruker SMART CCD diffractometer
21306 measured reflections
2571 independent reflections
1219 reflections with *I* > 2σ(*I*)
*R*_int_ = 0.115

#### Refinement

*R*[*F* ^2^ > 2σ(*F* ^2^)] = 0.072
*wR*(*F* ^2^) = 0.220
*S* = 1.03
2571 reflections
166 parameters
H atoms treated by a mixture of independent and constrained refinement
Δρ_max_ = 0.16 e Å^−3^
Δρ_min_ = −0.20 e Å^−3^

### 

Data collection: *SMART* (Bruker, 1997 ▶); cell refinement: *SAINT* (Bruker, 1997 ▶); data reduction: *SAINT*; program(s) used to solve structure: *SHELXS97* (Sheldrick, 2008 ▶); program(s) used to refine structure: *SHELXL97* (Sheldrick, 2008 ▶); molecular graphics: *SHELXTL* (Sheldrick, 2008 ▶); software used to prepare material for publication: *SHELXTL*.

## Supplementary Material

Crystal structure: contains datablock(s) global, I. DOI: 10.1107/S1600536812016248/hb6706sup1.cif

Structure factors: contains datablock(s) I. DOI: 10.1107/S1600536812016248/hb6706Isup2.hkl

Supplementary material file. DOI: 10.1107/S1600536812016248/hb6706Isup3.cml

Additional supplementary materials:  crystallographic information; 3D view; checkCIF report

## Figures and Tables

**Table 1:** Hydrogen-bond geometry (Å, °)

*D*—H⋯*A*	*D*—H	H⋯*A*	*D*⋯*A*	*D*—H⋯*A*
N2—H2*A*⋯O1^i^	0.89 (3)	2.08 (3)	2.935 (3)	162 (3)

Symmetry code: (i) 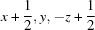.

**Fig. 1.**
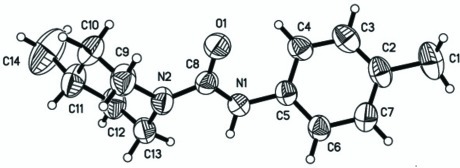
The structure of the title compound showing 30% probability displacement ellipsoids.

## supplementary crystallographic information

### Comment

Some compounds which are related to the title compound have been shown to have medicinal properties (Yang *et al.*, 1997). The structure of the title compound is shown in Fig. 1. The six-membered ring (C2—C6/N1) has a chair conformation. The bond lengths and angles can be compared to those within a related structure (Li, 2011). In the crystal, the molecules are linked into [100] chains by way of N—H···O hydrogen bonds.

### Experimental

A mixture of 4-methylpiperidine (0.08 mol), and *p*-tolylcarbamic chloride (0.08 mol) was stirred in refluxing ethanol (18 ml) for 4 h to afford the title compound (0.056 mol, yield 70%). Colourless blocks of the title compound were obtained by recrystallization from ethanol at room temperature.

### Refinement

H atoms were fixed geometrically and allowed to ride on their attached atoms, with C—H distances = 0.93–0.97 Å; N—H = 0.86 Å and with *U*_iso_(H) = 1.2*U*_eq_(C,*N*).

### Crystal data

**Table:**

C_14_H_20_N_2_O	*F*(000) = 1008
*M**_r_* = 232.32	*D*_x_ = 1.085 Mg m^−^^3^
Orthorhombic, *P**b**c**a*	Mo *K*α radiation, λ = 0.71073 Å
Hall symbol: -P 2ac 2ab	Cell parameters from 1979 reflections
*a* = 9.6192 (19) Å	θ = 3.1–27.5°
*b* = 11.127 (2) Å	µ = 0.07 mm^−^^1^
*c* = 26.574 (5) Å	*T* = 293 K
*V* = 2844.3 (9) Å^3^	Block, colorless
*Z* = 8	0.25 × 0.20 × 0.18 mm

### Data collection

**Table:**

Bruker SMART CCD diffractometer	1219 reflections with *I* > 2σ(*I*)
Radiation source: fine-focus sealed tube	*R*_int_ = 0.115
Graphite monochromator	θ_max_ = 25.3°, θ_min_ = 3.1°
φ and ω scans	*h* = −11→10
21306 measured reflections	*k* = −13→13
2571 independent reflections	*l* = −31→31

### Refinement

**Table:**

Refinement on *F*^2^	Primary atom site location: structure-invariant direct methods
Least-squares matrix: full	Secondary atom site location: difference Fourier map
*R*[*F*^2^ > 2σ(*F*^2^)] = 0.072	Hydrogen site location: inferred from neighbouring sites
*wR*(*F*^2^) = 0.220	H atoms treated by a mixture of independent and constrained refinement
*S* = 1.03	*w* = 1/[σ^2^(*F*_o_^2^) + (0.1032*P*)^2^ + 0.1575*P*] where *P* = (*F*_o_^2^ + 2*F*_c_^2^)/3
2571 reflections	(Δ/σ)_max_ < 0.001
166 parameters	Δρ_max_ = 0.16 e Å^−^^3^
0 restraints	Δρ_min_ = −0.20 e Å^−^^3^

### Special details

**Table:**

Geometry. All e.s.d.'s (except the e.s.d. in the dihedral angle between two l.s. planes) are estimated using the full covariance matrix. The cell e.s.d.'s are taken into account individually in the estimation of e.s.d.'s in distances, angles and torsion angles; correlations between e.s.d.'s in cell parameters are only used when they are defined by crystal symmetry. An approximate (isotropic) treatment of cell e.s.d.'s is used for estimating e.s.d.'s involving l.s. planes.
Refinement. Refinement of *F*^2^ against ALL reflections. The weighted *R*-factor *wR* and goodness of fit *S* are based on *F*^2^, conventional *R*-factors *R* are based on *F*, with *F* set to zero for negative *F*^2^. The threshold expression of *F*^2^ > σ(*F*^2^) is used only for calculating *R*-factors(gt) *etc*. and is not relevant to the choice of reflections for refinement. *R*-factors based on *F*^2^ are statistically about twice as large as those based on *F*, and *R*- factors based on ALL data will be even larger.

### Fractional atomic coordinates and isotropic or equivalent isotropic displacement parameters (Å^2^)

**Table:**

	*x*	*y*	*z*	*U*_iso_*/*U*_eq_	
N2	0.3590 (3)	0.2180 (2)	0.24983 (9)	0.0723 (7)	
O1	0.1457 (2)	0.2820 (2)	0.27689 (8)	0.0937 (8)	
N1	0.3387 (3)	0.3504 (2)	0.31682 (9)	0.0835 (8)	
C8	0.3092 (3)	0.1515 (3)	0.20795 (11)	0.0692 (8)	
C7	0.2741 (3)	0.2829 (3)	0.28129 (11)	0.0713 (8)	
C13	0.3740 (3)	0.1655 (3)	0.16191 (12)	0.0774 (9)	
H13A	0.4486	0.2180	0.1586	0.093*	
C12	0.3270 (4)	0.1007 (3)	0.12054 (12)	0.0900 (10)	
H12A	0.3714	0.1109	0.0897	0.108*	
C9	0.2002 (3)	0.0699 (3)	0.21213 (12)	0.0828 (10)	
H9A	0.1574	0.0577	0.2431	0.099*	
C11	0.2162 (4)	0.0217 (3)	0.12365 (14)	0.0927 (11)	
C5	0.4786 (4)	0.3297 (5)	0.33540 (14)	0.0885 (11)	
C10	0.1556 (4)	0.0073 (3)	0.17043 (16)	0.0964 (11)	
H10A	0.0822	−0.0464	0.1739	0.116*	
C14	0.1648 (5)	−0.0461 (4)	0.07747 (15)	0.1356 (17)	
H14A	0.2195	−0.0238	0.0488	0.203*	
H14B	0.0691	−0.0264	0.0714	0.203*	
H14C	0.1732	−0.1310	0.0832	0.203*	
C4	0.2562 (4)	0.4264 (4)	0.35029 (14)	0.1071 (13)	
H4A	0.1637	0.4367	0.3364	0.129*	
H4B	0.2990	0.5051	0.3529	0.129*	
C2	0.3901 (5)	0.3450 (4)	0.42415 (14)	0.1255 (15)	
H2B	0.4366	0.4221	0.4299	0.151*	
C6	0.4752 (4)	0.2737 (4)	0.38700 (14)	0.1118 (13)	
H6A	0.4370	0.1933	0.3844	0.134*	
H6B	0.5696	0.2667	0.3995	0.134*	
C3	0.2461 (4)	0.3699 (4)	0.40226 (15)	0.1223 (15)	
H3A	0.1945	0.2951	0.4000	0.147*	
H3B	0.1957	0.4234	0.4245	0.147*	
C1	0.3789 (9)	0.2780 (8)	0.47512 (19)	0.254 (4)	
H1A	0.4703	0.2653	0.4886	0.381*	
H1B	0.3341	0.2019	0.4701	0.381*	
H1C	0.3254	0.3254	0.4983	0.381*	
H2A	0.448 (3)	0.241 (3)	0.2491 (11)	0.088 (10)*	
H5A	0.532 (3)	0.280 (3)	0.3091 (13)	0.101 (10)*	
H5B	0.525 (4)	0.399 (4)	0.3365 (12)	0.115 (15)*	

### Atomic displacement parameters (Å^2^)

**Table:**

	*U*^11^	*U*^22^	*U*^33^	*U*^12^	*U*^13^	*U*^23^
N2	0.0617 (16)	0.0876 (18)	0.0677 (16)	−0.0074 (14)	−0.0034 (13)	−0.0075 (14)
O1	0.0612 (14)	0.136 (2)	0.0839 (16)	0.0009 (13)	−0.0030 (11)	−0.0044 (13)
N1	0.0641 (16)	0.114 (2)	0.0720 (17)	0.0103 (14)	−0.0053 (13)	−0.0237 (16)
C8	0.0735 (19)	0.0647 (19)	0.069 (2)	−0.0026 (16)	−0.0056 (15)	0.0013 (15)
C7	0.0629 (19)	0.085 (2)	0.0656 (19)	−0.0019 (17)	0.0031 (16)	0.0086 (17)
C13	0.093 (2)	0.0668 (19)	0.073 (2)	−0.0078 (17)	−0.0045 (17)	−0.0032 (16)
C12	0.124 (3)	0.075 (2)	0.071 (2)	0.006 (2)	−0.012 (2)	−0.0020 (18)
C9	0.086 (2)	0.075 (2)	0.087 (2)	−0.0111 (18)	−0.0064 (18)	0.0081 (18)
C11	0.113 (3)	0.067 (2)	0.099 (3)	0.004 (2)	−0.034 (2)	−0.0082 (19)
C5	0.071 (2)	0.117 (3)	0.078 (2)	−0.007 (2)	0.0001 (17)	−0.025 (2)
C10	0.100 (3)	0.068 (2)	0.121 (3)	−0.0100 (18)	−0.027 (2)	−0.002 (2)
C14	0.170 (4)	0.111 (3)	0.126 (3)	−0.005 (3)	−0.058 (3)	−0.031 (3)
C4	0.090 (2)	0.139 (3)	0.093 (3)	0.023 (2)	−0.002 (2)	−0.031 (2)
C2	0.142 (4)	0.166 (4)	0.069 (2)	0.028 (3)	0.000 (2)	−0.010 (2)
C6	0.113 (3)	0.130 (3)	0.092 (3)	0.020 (3)	−0.017 (2)	−0.014 (2)
C3	0.108 (3)	0.161 (4)	0.097 (3)	0.006 (3)	0.029 (2)	−0.031 (3)
C1	0.344 (11)	0.333 (10)	0.084 (4)	0.090 (8)	0.026 (5)	0.042 (5)

### Geometric parameters (Å, º)

**Table:**

N2—C7	1.374 (4)	C5—H5B	0.89 (4)
N2—C8	1.420 (4)	C10—H10A	0.9300
N2—H2A	0.89 (3)	C14—H14A	0.9600
O1—C7	1.241 (3)	C14—H14B	0.9600
N1—C7	1.357 (4)	C14—H14C	0.9600
N1—C4	1.462 (4)	C4—C3	1.521 (5)
N1—C5	1.452 (4)	C4—H4A	0.9700
C8—C13	1.382 (4)	C4—H4B	0.9700
C8—C9	1.391 (4)	C2—C6	1.508 (5)
C13—C12	1.390 (4)	C2—C3	1.528 (6)
C13—H13A	0.9300	C2—C1	1.550 (6)
C12—C11	1.384 (5)	C2—H2B	0.9800
C12—H12A	0.9300	C6—H6A	0.9700
C9—C10	1.378 (4)	C6—H6B	0.9700
C9—H9A	0.9300	C3—H3A	0.9700
C11—C10	1.382 (5)	C3—H3B	0.9700
C11—C14	1.524 (5)	C1—H1A	0.9600
C5—C6	1.506 (5)	C1—H1B	0.9600
C5—H5A	1.03 (3)	C1—H1C	0.9600
			
C7—N2—C8	123.4 (3)	H14A—C14—H14B	109.5
C7—N2—H2A	116 (2)	C11—C14—H14C	109.5
C8—N2—H2A	117 (2)	H14A—C14—H14C	109.5
C7—N1—C4	119.7 (3)	H14B—C14—H14C	109.5
C7—N1—C5	125.0 (3)	N1—C4—C3	110.4 (3)
C4—N1—C5	112.8 (3)	N1—C4—H4A	109.6
C13—C8—C9	119.0 (3)	C3—C4—H4A	109.6
C13—C8—N2	118.9 (3)	N1—C4—H4B	109.6
C9—C8—N2	122.1 (3)	C3—C4—H4B	109.6
O1—C7—N1	121.7 (3)	H4A—C4—H4B	108.1
O1—C7—N2	122.0 (3)	C6—C2—C3	109.8 (3)
N1—C7—N2	116.2 (3)	C6—C2—C1	110.9 (4)
C8—C13—C12	119.7 (3)	C3—C2—C1	110.9 (4)
C8—C13—H13A	120.2	C6—C2—H2B	108.4
C12—C13—H13A	120.2	C3—C2—H2B	108.4
C13—C12—C11	122.2 (3)	C1—C2—H2B	108.4
C13—C12—H12A	118.9	C2—C6—C5	112.9 (3)
C11—C12—H12A	118.9	C2—C6—H6A	109.0
C10—C9—C8	120.0 (3)	C5—C6—H6A	109.0
C10—C9—H9A	120.0	C2—C6—H6B	109.0
C8—C9—H9A	120.0	C5—C6—H6B	109.0
C10—C11—C12	116.9 (3)	H6A—C6—H6B	107.8
C10—C11—C14	122.0 (4)	C4—C3—C2	111.3 (3)
C12—C11—C14	121.1 (4)	C4—C3—H3A	109.4
N1—C5—C6	110.8 (3)	C2—C3—H3A	109.4
N1—C5—H5A	108.7 (18)	C4—C3—H3B	109.4
C6—C5—H5A	113.7 (18)	C2—C3—H3B	109.4
N1—C5—H5B	110 (2)	H3A—C3—H3B	108.0
C6—C5—H5B	110 (2)	C2—C1—H1A	109.5
H5A—C5—H5B	104 (3)	C2—C1—H1B	109.5
C9—C10—C11	122.2 (3)	H1A—C1—H1B	109.5
C9—C10—H10A	118.9	C2—C1—H1C	109.5
C11—C10—H10A	118.9	H1A—C1—H1C	109.5
C11—C14—H14A	109.5	H1B—C1—H1C	109.5
C11—C14—H14B	109.5		

### Hydrogen-bond geometry (Å, º)

**Table:**

*D*—H···*A*	*D*—H	H···*A*	*D*···*A*	*D*—H···*A*
N2—H2*A*···O1^i^	0.89 (3)	2.08 (3)	2.935 (3)	162 (3)

Symmetry code: (i) *x*+1/2, *y*, −*z*+1/2.
